# Pharmacokinetics and Monitoring of Methotrexate in Adults with Acute Lymphoblastic Leukaemia: A 10-Year Follow-Up at an Italian Centre

**DOI:** 10.3390/jcm14207400

**Published:** 2025-10-20

**Authors:** Pasquale Fabio Calabrò, Letizia Biso, Marianna Lastella, Arianna Bandini, Marta Banchi, Costanza Tacchi, Donghao Tang, Marco Carli, Stefano Fogli, Aldo Paolicchi, Marco Scarselli, Antonello Di Paolo, Guido Bocci

**Affiliations:** 1 Department of Translational Research and New Technologies in Medicine and Surgery, University of Pisa, 56126 Pisa, Italy; calabro.pf@gmail.com (P.F.C.); l.biso@studenti.unipi.it (L.B.); arianna.bandini@phd.unipi.it (A.B.); marta.banchi@phd.unipi.it (M.B.); costanza.tacchi@phd.unipi.it (C.T.); donghao.tang@phd.unipi.it (D.T.); carlimarco@outlook.it (M.C.); aldo.paolicchi@unipi.it (A.P.); marco.scarselli@unipi.it (M.S.); 2 Unit of Clinical Pharmacology and Pharmacogenetics, Azienda Ospedaliero-Universitaria Pisana, 56126 Pisa, Italy; marianna.lastella@ao-pisa.toscana.it (M.L.); stefano.fogli@unipi.it (S.F.); antonello.dipaolo@unipi.it (A.D.P.); 3 Department of Clinical and Experimental Medicine, University of Pisa, 56126 Pisa, Italy

**Keywords:** methotrexate, therapeutic drug monitoring, acute lymphoblastic leukaemia, renal function

## Abstract

**Background**: High-dose methotrexate (HDMTX) is widely used for acute lymphoblastic leukaemia (ALL), but its pharmacokinetic (PK) variability and toxicity require therapeutic drug monitoring (TDM). Our 10-year retrospective study investigated HDMTX PK parameters and their associations with renal and hepatic biomarkers in an Italian cohort of adult patients with ALL. **Methods**: Plasma MTX concentrations [MTX C(p)] were measured at 24-, 48-, and 72 h post-infusion. PK modelling was performed to calculate area under the curve (AUC_0 → 72 h_) and half-life (t½). Creatinine, total bilirubin, and sample quality indices were retrieved from routine clinical laboratory analyses. **Results**: Mean (±SEM) MTX plasma concentrations were 36.09 ± 15.53 μmol/L, 0.93 ± 0.43 μmol/L, and 0.30 ± 0.07 μmol/L at 24, 48, and 72 h, respectively, with marked inter-patient variability. PK analysis showed a mean AUC_0 → 72 h_ of 112.85 ± 34.09 h·μmol/L and a t½ of 17.15 ± 2.40 h. MTX C(p) and AUC_0 → 72 h_ showed significant positive correlations with serum creatinine at all time points, confirming renal function as a major MTX clearance determinant. Age moderated the relationship at 72 h, with younger patients showing stronger correlations. Hepatic function measured by total bilirubin also correlated with MTX C(p) and AUC_0 → 72 h_ at 48 and 72 h, especially in younger patients, suggesting a hepatic contribution to MTX variability. No associations were found between the PK parameters and lipemic, icterus, or haemolysis indices. **Conclusions**: These findings highlight the value of integrating renal and hepatic biomarkers into HDMTX drug monitoring protocols. Such biomarker-informed TDM may improve the safety and efficacy by identifying patients at risk of delayed clearance and toxicity, especially younger individuals or those with renal insufficiency.

## 1. Introduction

Methotrexate (MTX) is an antimetabolite that exerts its main antineoplastic activity throughout the inhibition of dihydrofolate reductase, therefore inhibiting the formation of tetrahydrofolate, the active form of folic acid, required for the synthesis of the nucleoside thymidine [1]. MTX has been used for the treatment of acute leukaemia since the 1950s [2]. In the following decades, its use has expanded to include the treatment of solid tumours and other rheumatological, dermatological, and gynaecological conditions, both in children and in adults [3,4,5,6].

Acute lymphoblastic leukaemia (ALL) is the most common form of cancer in children and younger adults, but it can also occur in older individuals, posing even more challenges in terms of treatment [7,8]. MTX is widely used in ALL as part of induction/consolidation regimens and for central nervous system (CNS) prophylaxis or treatment. In this context, MTX is used for cytotoxic effect against rapidly dividing lymphoblasts and for CNS-directed therapy; administration routes include intravenous, intrathecal, and oral or subcutaneous dosing depending on intent and protocol [9]. High-dose MTX (HDMTX), defined as a >500 mg/m^2^ intravenous dose, still remains one of the key treatments for ALL; however, this regime can expose patients to toxicity risks, especially at the renal level [7,10]. Another important issue with HDMTX treatment is the heterogeneity in pharmacokinetics (PK) among different patients [11].

High PK variability in the population and the risk of toxicity are the main reasons for therapeutic drug monitoring (TDM) during HDMTX treatment, and the importance of TDM in patients with ALL is well-established [12]. In 2022, the Chinese Pharmacological Society implemented new evidence-based guidelines for MTX monitoring, recommending routine TDM for this medication at 24, 48, and 72 h after MTX administration, or until the MTX plasmatic concentration [C(p)] is less than 0.1–0.2 μmol/L [13]. Regarding the MTX C(p) range for ALL, data from existing research mostly come from paediatric populations, suggesting that MTX should be in the 16–40 μmol/L range in children at 24 h to reduce the risk of toxicity and achieve an adequate response. Similarly, the concentration after 48 h should be below 1 μmol/L, and after 72 h, it should become less than 0.1–0.2 μmol/L [14]. Unfortunately, the adult population with ALL is understudied, but research on other kinds of cancer has confirmed an increased risk of adverse events when the MTX C(p) is >1 μmol/L at 48 h [14]. The guidelines for HDMTX monitoring also recommend testing kidney function before and during treatment. Renal damage is not uncommon during MTX treatment, and it is most probably due to MTX or 7-hydroxy-MTX precipitation in the kidney tubules or by direct toxicity of MTX [15]. MTX dose adjustments should be considered for creatinine clearance under 100 mL/min to reduce the risk of adverse drug reactions [13].

The rationale for liver function monitoring is supported by lower quality evidence, but it is still recommended [13]. It is known that MTX can lead to hepatotoxicity, especially in the presence of renal comorbidities or drug–drug interactions, and patients may develop an increase in transaminase or bilirubin values [10,16].

With these premises, the aim of our study was to find associations between the PK parameters and other laboratory data to identify potential early toxicity indicators in the specific population of adult patients with ALL.

## 2. Materials and Methods

### 2.1. Study Sample

All blood samples were collected from routine analyses at the Haematology Unit of the University Hospital of Pisa (Azienda Ospedaliero Universitaria Pisana, AOUP) from 2013 to 2022 and came from the inpatient ward. Samples were then analysed at the Unit of Clinical Pharmacology and Pharmacogenetics and the Unit of Clinical Chemical Analysis Laboratory of the University Hospital of Pisa. All patients were over 18 years old and followed an HDMTX intravenous regime with a 500–4500 mg/m^2^ dose range. In total, 521 samples were collected from 106 patients (64 males and 42 females) with ALL. Of these patients, the majority had a de novo diagnosis; however, ten males and nine females were treated again with HDMTX for relapses. Unfortunately, information on the patients’ clinical status or on concomitant treatment could not be retrieved.

### 2.2. Analytical Determination of MTX Plasmatic Concentrations

Two mL blood samples were collected; blood was centrifuged for 15 min at 3000 rpm and plasma was isolated before analysis. Plasma MTX drug levels were estimated by using the Abbott ARCHITECT Methotrexate Assay I2000SR (Abbott Diagnostics, Chicago, IL, USA) [17]. The ARCHITECT Methotrexate assay is utilised in clinical laboratories for the quantitative determination of methotrexate in human serum or plasma using chemiluminescent microparticle immunoassay (CMIA) technology. The low and high limits of concentration of the Abbott ARCHITECT Methotrexate Assay Kit were 0.040 and 1.500 μmol/L, respectively. Samples with an MTX concentration higher than 1.500 μmol/L were diluted following the manual dilution procedure provided by the manufacturer. The ARCHITECT Methotrexate assay is a one-step competitive, microparticle immunoassay. A sample of anti-MTX coated paramagnetic microparticles and MTX acridinium-labelled conjugate is added to the reaction vessel. MTX present in the sample and the MTX-conjugate compete for binding on the anti-MTX antibodies. The resulting chemiluminescent reaction, measured as relative light units, is inversely proportional to the MTX concentration of the sample. The test imprecision was acceptable, with the within-run coefficient of variation (CV) being <4% and between-run CV < 8%. Linearity was evaluated covering the range 0.083–1.157 µmol/L of MTX.

As for the laboratory measures, we retrieved the creatinine and total bilirubin concentrations. Moreover, we obtained information on the lipemic, icterus, and haemolysis index values. These indices are the most common pre-analytical factors that can impact routine clinical tests results, and they indicate the presence of high plasmatic concentrations of triglycerides, bilirubin, and haemoglobin, respectively [18]. All of these data were obtained from routinary laboratory tests and then registered in the Openlis software database (v. 21.1.0; Engineering Ingegneria Informatica S.p.A., Roma, Italy) as well as the serum MTX levels. This database can be accessed only by authorised personnel by a recognition card and a password, ensuring the patients’ privacy. The activity of our laboratory is ISO9001 certified [19], and quality control is regularly tested with external samples.

### 2.3. Data Analysis

Data samples were reported for different subgroups (post-treatment days, gender, age). MTX C(p) concentrations were determined at 24, 48, and 72 h after HDMTX infusion, and were expressed in µmol/L.

Pharmacokinetic analyses were carried out with PKSolver, an add-in program for pharmacokinetic and pharmacodynamic data analysis in Microsoft Excel [20]. Individual MTX concentration versus time datasets were fitted according to a one-compartment model, using nonlinear least-squares regression analysis. The Cmax was identified from the inspection of MTX concentration–time plots. The t½ was obtained from the equation t½ = 0.693/k, where k is the slope of the exponential. The AUC was calculated by the trapezoidal method for the area from time 0 (t0) to the time of last measurable concentration or last blood sample (72 h).

The patients’ pharmacokinetic parameters were reported as the mean ± SE, and Kruskal–Wallis or ANOVA tests were carried out, after performing the Kolmogorov–Smirnov or Shapiro–Wilk normality test, to analyse the differences among subgroups. If only two subgroups were present, the Mann–Whitney U test was used. Correlations between MTX C(p), AUC, or t½, and biological parameters were calculated with the Spearman rank correlation test. When feasible, moderation analysis was carried out in search of significant moderators (i.e., age, gender). The Friedman test was used to analyse repeated measures of plasma concentrations in the same patient among three points h. The level of significance was set at *p* < 0.05. The graphs, descriptive analysis, ANOVAs, Kruskal–Wallis, Mann–Whitney, Spearman, Friedman, and moderation tests were performed using either GraphPad Prism 7.05 for Windows (GraphPad Software, San Diego, CA, USA) or JASP (JASP Team, 2024; version 0.18.3.0) software. Graphs were created using R (version 4.3.2) and the ggplot2 package [21].

### 2.4. Ethical Statement

All patients gave their consent to the collection of samples for routine MTX TDM and for routine biochemical analyses as well as the subsequent use of their data for research. Patient data were then rendered anonymous for the different analyses. The study was conducted in accordance with the Declaration of Helsinki, and the protocol was approved by the Ethics Committee of the Area Vasta Nord Ovest (CEAVNO) (Project identification code no. CEAVNO_Bocci_02-02-2023).

## 3. Results

### 3.1. Descriptive Analyses of MTX Samples and Patients During the Period 2013–2022

Among the 521 MTX C(p) collected samples, we retrieved 169, 128, and 96 samples measuring the MTX C(p) levels after 24, 48, and 72 h of treatment, respectively. The remaining 128 samples corresponded to samples obtained after 72 h from treatment and were not included in our analysis. Of the included 393 samples, 239 were from 64 male patients, whereas 154 samples came from 42 female patients. The average age for males at the time of treatment was 47.58 (range 19–76) years, while for females it was 49.11 (range 19–72) years.

**Figure 1:**
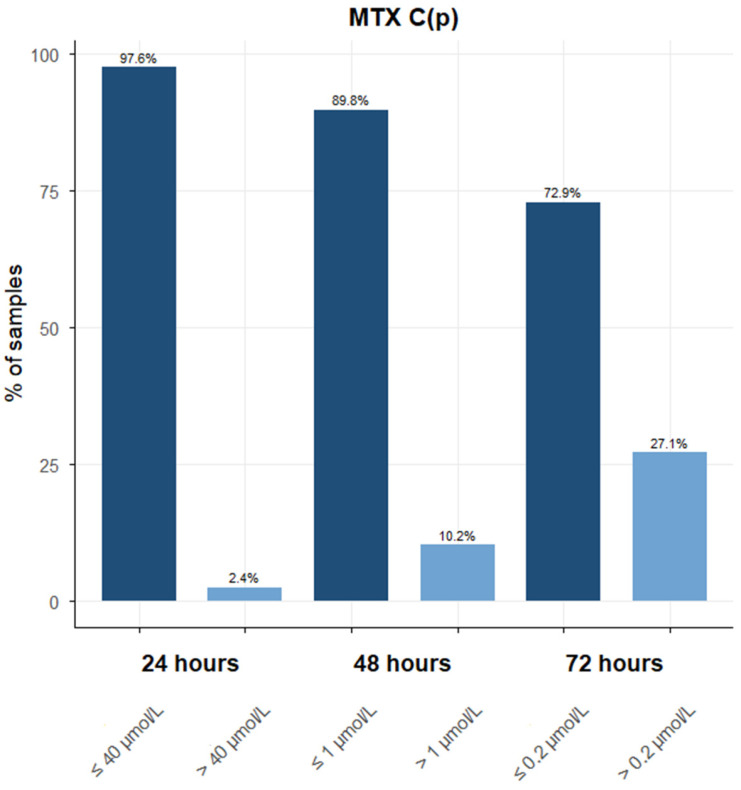
Distribution of the total number of MTX C(p) values retrieved at 24, 48, and 72 h, with a focus on samples with possible toxic concentrations based on data reported by the scientific literature. At 24 h, 165 samples had an MTX C(p) ≤ 40 μmol/L, and only four samples showed toxic concentrations above 40 μmol/L. At 48 h, 115 samples had an MTX C(p) ≤ 1 μmol/L, and 13 were >1 μmol/L. Finally, at 72 h, 70 samples had an MTX C(p) ≤ 0.2 μmol/L, and 26 were above 0.2 μmol/L.

The overall mean value (±SEM) for MTX C(p) at 24 h was 36.09 (±15.53) μmol/L, with a difference (although not statistically significant) between males, 51.93 (±26.82) μmol/L and females, 18.00 (±12.02) μmol/L. At 48 h, the average concentration was 0.93 (±0.43) μmol/L, 1.24 (±0.67) μmol/L for males and 0.39 (±0.09) μmol/L for females. Thirteen samples presented an MTX C(p) higher than 1 μmol/L. Finally, the average MTX C(p) values at 72 h were 0.30 (±0.07) μmol/L, with males presenting an average of 0.3 (±0.09) μmol/L, and females an average of 0.28 (±0.07) μmol/L. Twenty-six samples were above 0.2 μmol/L at 72 h, as shown in Figure 1. A summary of the descriptive results is summarised in Table 1.

We analysed the possible presence of correlations between the MTX C(p) values and the patients’ age. We found no direct correlation between age and the MTX C(p) at 24 (ρ = 0.048, *p* = 0.536), 48 (ρ = 0.001, *p* = 0.991), or 72 h (ρ = −0.088, *p* = 0.396). Most of the patients completed one MTX TDM after the first cycle of treatment. In 44 cases (41.5%), patients completed at least two MTX TDM after the second cycle. Among them, five completed three (4.7%), three patients completed four (2.8%), and two patients (1.9%) completed five MTX TDM after respective therapeutic cycles.

### 3.2. PK Analysis

Based on the MTX C(p) at 24, 48, and 72 h, we calculated the AUC_0 → 72 h_ and t½. The mean AUC (±SEM) was 112.85 h·μmol/L (±34.09), with men showing a higher mean AUC of 136.43 h·μmol/L (±50.37) compared with women, which had an average value of 68.77 h·μmol/L (±26.12). Considering only values from the first cycle of treatment, the average AUC was 98.48 (±34.83), 110.48 (50.50) for males, and 77.18 h·μmol/L (±36.98) for females. At the second cycle, the average AUC was 126.86 h·μmol/L (±89.10), with a difference between males, which showed an average AUC of 165.21 h·μmol/L (±131.07), and females, which had a mean AUC of 46.33 h·μmol/L (±28.42). The mean AUC for the third cycle was 237.92 h·μmol/L (±200.64), higher for males (344.74 ± 336.72 h·μmol/L) than for females (75.87 ± 68.47 h·μmol/L). In general, we noticed a high heterogeneity between patients for AUC values. Although we reported an increase in the AUC values during cycles, there was no statistically significant difference between the AUC in the first, second, or third cycle of treatment. We then searched for AUC differences between the first and the second cycles in males and females that had completed both cycles of treatment, but we did not find any statistically significant difference. We did not include AUC values from the third cycle of treatment in the analysis due to the scarcity of patients.

Considering t½, the mean overall value (±SEM) was 17.15 (±2.40) h, 14.45 (±1.15) h for men and 22.20 (±6.54) h for women. At the first cycle, mean t½ was 17.96 (±3.60) h, 13.32 (±1.48) h for men and 26.20 (±9.57) h for women. The average t½ at the second cycle of treatment was 16.09 (±2.04) h, 15.91 (±2.46) h for men and 16.46 (±2.04) h for women. Finally, considering the few patients that had completed three cycles of treatment, we calculated a mean t½ of 12.88 (±2.48) h, 14.50 (±2.49) h for men and 10.44 (±5.23) for women. No statistically significant difference was detected between the first, second, and third cycle. Considering only the patients that had completed at least two cycles, there were no differences in terms of t½ at the first and second cycle for men compared with women. When analysing differences between cycles, there was a significant difference between t½ at the first compared with the second cycle for men calculated with the Student’s t-test (*p* = 0.004), with a higher mean t½ at the second cycle. Conversely, no difference was observed between t½ at the first versus the second cycle for women. A summary of the AUC and t½ parameters is shown in Table 1.

### 3.3. Correlations Between MTX C(p) and Biochemical Parameters

**Figure 2:**
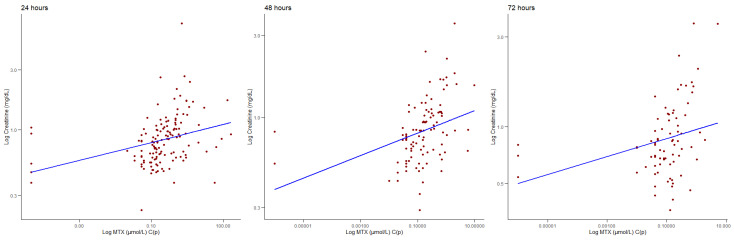
Correlations between the MTX (μmol/L) C(p) levels and creatinine (mg/dL) levels at 24, 48, and 72 h.

Among the 169, 128, and 96 MTX C(p) samples, corresponding to MTX C(p) levels at 24, 48, and 72 h, we retrieved creatinine levels of the same day for 137, 102, and 77 samples, respectively. Spearman’s correlations performed pairwise showed a positive correlation between MTX C(p) and creatinine levels at 24 (ρ = 0.463, *p* < 0.001), 48 (ρ = 0.546, *p* < 0.001), and 72 h (ρ = 0.415, *p* < 0.001), as shown in Figure 2. We then hypothesised the presence of moderators (age, gender) that could affect the correlation between MTX C(p) and creatinine. We found that age and gender did not affect the relationship between MTX C(p) and creatinine at 24 and 48 h; however, we did find a moderating effect of age at 72 h (z-value = −4.463, *p* < 0.001), with patients below the 50th age percentile showing a stronger correlation between creatinine levels and MTX C(p) values.

We then searched for possible correlations between MTX C(p) and the lipemic, icterus, and haemolysis indices at 24, 48, and 72 h. We did not find any correlation between MTX C(p) and the lipemic index at 24 (ρ = −0.086, *p* = 0.277), 48 (ρ = −0.065, *p* = 0.471), and 72 h (ρ = −0.012, *p* = 0.909). Similarly, there was no correlation between MTX C(p) and the icterus index at 24 (ρ = −0.085, *p* = 0.283), 48 (ρ = 0.113, *p* = 0.210), and 72 h (ρ = −0.086, *p* = 0.277). As for the correlation between MTX C(p) and the haemolysis index, we did not find statistically significant results at 24 (ρ = −0.098, *p* = 0.215), 48 (ρ = 0.006, *p* = 0.944), or 72 h (ρ = −0.125, *p* = 0.230).

**Figure 3:**
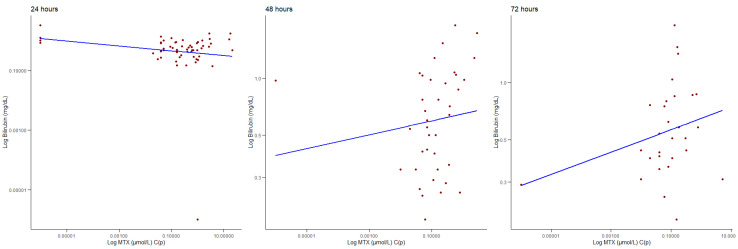
Correlation between MTX (μmol/L) C(p) and the bilirubin (mg/dL) levels at 24, 48, and 72 h.

Finally, regarding possible correlations between MTX C(p) and the total bilirubin, it is important to note that the total bilirubin was not routinely monitored in all patients, therefore we were able to retrieve 57 samples at 24 h, 39 samples at 48 h, and 29 samples at 72 h. We found no significant correlation at 24 h (ρ = −0.059, *p* = 0.667); however, we found a significant positive correlation between MTX C(p) and the total bilirubin levels at 48 h (ρ = 0.339, *p* = 0.035) and at 72 h (ρ = 0.074, *p* = 0.0481), as shown in Figure 3. We found no moderating effect of age or gender at 24 and 48 h. We found a moderating effect of age at 72 h (z-value = 2.133, *p* = 0.033), so for younger patients (at the 16th age percentile), there was a stronger relationship between the bilirubin levels and MTX C(p). Conversely, gender did not influence the MTX C(p) levels regarding the total bilirubin values at 72 h.

### 3.4. Correlations Between AUC_0 → 72 h_ and Biochemical Parameters

We then searched for correlations between the MTX AUC_0 → 72 h_ values and biochemical parameters. We considered AUC values regardless of the cycle of treatment, and we used values for creatinine, lipemic, icterus, haemolysis indices, and total bilirubin collected 24, 48, or 72 h after MTX treatment.

**Figure 4:**
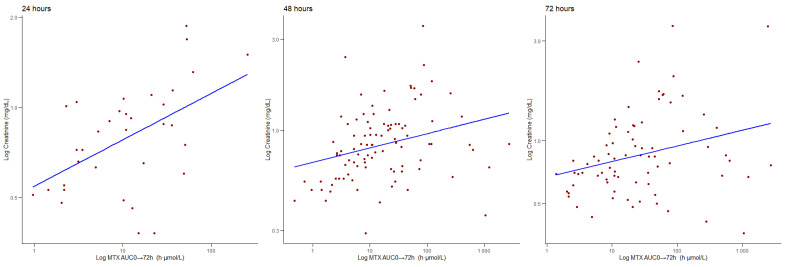
Correlations between the MTX AUC_0 → 72 h_ (h·μmol/L) values and creatinine (mg/dL) levels at 24, 48, and 72 h.

We found a positive correlation between AUC and creatinine at 24 (ρ = 0.518, *p* < 0.001), 48 (ρ = 0.375 *p* < 0.001), and 72 h (ρ = 0.327, *p* = 0.004), as can be observed in Figure 4. When searching for possible moderators for these results, we could not find a moderating effect of age or gender at 24 or 48 h. When considering creatinine levels at 72 h, we did not find gender as a moderator; however, we found a moderating effect of age (z-value = −2.533, *p* = 0.011), so for younger patients, there was a higher increase in MTX C(p) for higher creatinine levels.

Considering the lipemic index, we found no correlation with the AUC at 24 (ρ = 0.082, *p* = 0.378), 48 (ρ = 0.007, *p* = 0.941), or 72 h (ρ = −0–012, *p* = 0.909). Similarly, there was no correlation between the AUC and icterus index at 24 (ρ = 0.014, *p* = 0.884), 48 (ρ = 0.114, *p* = 0.219), or 72 h (ρ = 0.074, *p* = 0.481). Moreover, there was no correlation between the AUC and haemolysis index at 24 (ρ = −0.140, *p* = 0.133), 48 (ρ = 0.020, *p* = 0.827), or 72 h (ρ = −0.125, *p* = 0.230).

**Figure 5:**
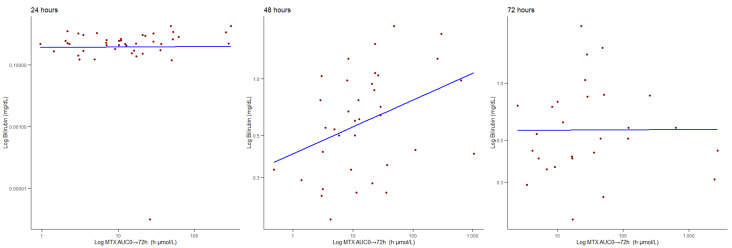
Correlations between the MTX AUC_0 → 72 h_ (h·μmol/L) values and bilirubin (mg/dL) levels at 24, 48, and 72 h.

Finally, we found a positive correlation between the AUC and total bilirubin at 24 (ρ = 0.341, *p* = 0.03), 48 (ρ = 0.365, *p* = 0.029), and 72 h (ρ = 0.372, *p* = 0.047) (Figure 5). When searching for possible moderators, we did not find an effect of age and gender at 24 and 48 h, however, there was an effect of age (but not gender) at 72 h (z-value = 5.257, *p* < 0.001), with patients under the 50th percentile of age presenting a stronger positive relationship between the bilirubin levels and AUC.

### 3.5. Correlations Between t½ and Biochemical Parameters

Finally, we searched for possible correlations between the t½ values, and the biochemical parameters collected at 24, 48, and 72 h.

We found a positive trend between t½ and creatinine at 24 (ρ = 0.176, *p* = 0.07) and 72 h (ρ = 0.223, *p* = 0.053), although it did not reach statistical significance. We found a positive correlation between t ½ and the creatinine values at 48 h (ρ = 0.205, *p* = 0.046). Age and gender did not influence this relationship.

There was no correlation between t½ and the lipemic index at 24 (ρ = −0.031, *p* = 0.741), 48 (ρ = −0.109, *p* = 0.239), and 72 h (ρ = −0.044, *p* = 0.672). Similarly, the t ½ did not correlate with the icterus index at 24 (ρ = −0.053, *p* = 0.569), 48 (ρ = 0.027, *p* = 0.768), or 72 h (ρ = −0.142, *p* = 0.176). We could not find significant correlations between t½ and the haemolysis index at 24 (ρ = −0.147, *p* = 0.115), 48 (ρ = −0.034, *p* = 0.717), or 72 h (ρ = −0.065, *p* = 0.533).

When considering the total bilirubin, we found no correlation between t½ and the bilirubin levels at 24 (ρ = 0.080 *p* = 0.625), 48 (ρ = 0.130, *p* = 0.448), or 72 h (ρ = 0.231, *p* = 0.237).

## 4. Discussion

In our retrospective study, we evaluated the PK properties of HDMTX and its correlations with clinical biochemical parameters. We observed marked inter-patient variability in plasma concentrations, AUC, and t½ across treatment cycles. While no significant differences were found by age or gender overall, a male-specific increase in t½ between the first and second cycle was noted. MTX exposure (C(p) and AUC) correlated strongly with creatinine at all time points, underscoring the central role of renal clearance, with additional age-dependent effects at later time points. This relationship likely reflects MTX predominant renal elimination through glomerular filtration and active tubular secretion mediated by organic anion transporters (OAT1 and OAT3). Impaired transporter activity or decreased glomerular filtration with age may therefore prolong MTX half-life and increase systemic exposure [22]. Total bilirubin also showed moderate correlations with the MTX levels and AUC, particularly in younger patients, whereas the lipemic, icterus, and haemolysis indices showed no influence. The observed bilirubin association could be related to competitive hepatic transport and metabolism, as MTX and bilirubin share efflux pathways via multidrug resistance-associated proteins (MRPs) and organic anion transporting polypeptides (OATPs). In younger individuals, higher hepatic metabolic activity may accentuate such interactions, explaining the age-dependent pattern [23].

Interestingly, we could not find statistically significant differences between male and female patients, nor a direct correlation between MTX C(p) and age. A recent systematic PK analysis in patients undergoing MTX treatment for oncologic diagnoses identified gender as a significant PK factor, with lower clearance in females receiving HDMTX compared with males [24]. As per age differences, a comprehensive PK analysis in both paediatric and adult patients with different cancer types showed that age per se did not independently predict the MTX levels [25].

We observed high inter-patient variability in AUC_0 → 72 h_ values for MTX, consistent with previous studies on both paediatric and adult populations, highlighting the influence of renal function, hydration, and other individual factors [10,15,26]. At the molecular level, such variability is plausibly driven by genetic polymorphisms in drug transporter genes (e.g., SLC19A1, ABCG2, ABCC2, ABCC4) and genes involved in folate metabolism. These variants may influence MTX uptake, intracellular retention via polyglutamation, efflux, and elimination, thus altering effective exposure [27]. The lack of statistically significant changes in the AUC between cycles is consistent with findings on patients with osteosarcoma treated with MTX [28]; however, we also must consider the relatively small sample size of our study, which could have also influenced our results.

We did not observe any significant difference in MTX t½ across consecutive treatment cycles when considering the full patient cohort. This is consistent with prior studies showing that MTX t½ remains relatively stable across cycles in the absence of major renal or metabolic shifts [15]. However, when analysing patients who completed at least two cycles, a statistically significant increase in t½ was detected in males between the first and second cycle, while no such difference was observed in females. The biological basis for this male-specific increase is unclear, but previous studies have suggested that gender-related differences in MTX clearance may be modest but measurable [29,30]. The absence of a change in female patients may indicate a more stable PK profile across cycles, although further investigation with larger sample sizes is needed to validate this trend.

Consistently, MTX plasma concentrations C(p) were positively correlated with creatinine at 24, 48, and 72 h. This finding is supported by studies in paediatric patients with ALL showing that elevated creatinine impairs MTX clearance [31]. The correlation between MTX C(p) and creatinine levels has also been observed in a few retrospective studies, mostly conducted in East Asian populations. In these studies, researchers found a positive correlation between the creatinine levels and MTX C(p) in both paediatric and adult patients with ALL and other haematologic cancers [32,33,34]. There is, however, a lack of information regarding other populations that may carry different polymorphisms that could potentially show different results.

Similarly, AUC_0 → 72 h_ showed significant positive correlations with creatinine at all time points, indicating that renal clearance is the primary determinant of MTX exposure. Our finding of a stronger AUC–creatinine relationship at 72 h in younger patients suggests an age-dependent renal management of MTX, in line with paediatric population models [25,35]. The MTX elimination t½ also showed a moderate correlation with creatinine at 48 h, with non-significant trends at 24 and 72 h. This supports earlier evidence that reduced renal function prolongs MTX elimination [33,34]. We found no moderating effect of age or gender on this relationship, though Kristensen et al. did report age-associated increases in t½, possibly masked in our more homogeneous age sample [36].

Across all time points, no correlation was found between MTX C(p), AUC_0 → 72 h_, or t½ and the lipemic, icterus, or haemolysis indices. This is consistent with assay validation studies demonstrating that these sample quality markers do not interfere with MTX quantification [37,38,39]. Furthermore, most of the literature on MTX PK highlights renal and hepatic influences rather than pre-analytical matrix factors, underscoring the robustness of clinical MTX assays and the specificity of renal biomarkers in guiding interpretation.

We found moderate positive correlations between the total bilirubin at 48 and 72 h and both MTX C(p) and AUC_0 → 72 h_. A stronger association in younger patients at 72 h suggests age-related differences in hepatic metabolism. A correlation between MTX C(p) and bilirubin levels has been described in recent years in a retrospective study in a Chinese paediatric population with ALL, confirming previous preclinical findings, but the scientific data on the adult population with ALL are scarce. Moreover, elevated bilirubin has been associated with hepatic metabolite accumulation and impaired MTX clearance in patients with osteosarcoma [40,41,42]. These results imply that hepatic metabolism, particularly in younger patients, may meaningfully contribute to MTX exposure variability [28]. In contrast, t½ did not correlate significantly with bilirubin at any time point, suggesting that while bilirubin may reflect the hepatic metabolism of MTX, it does not directly influence the elimination kinetics, which remain primarily renal-dependent.

Notwithstanding the very interesting real-world data, our study presents some limitations. First, its retrospective design restricts the ability to control for confounding variables and limits causal inferences. Second, the relatively small sample size may reduce statistical power and increase the risk of type II errors, particularly in subgroup or moderation analyses. Third, we could not retrieve access to clinical data such as the patients’ clinical outcomes, hydration status, concomitant medications, toxicity, or disease severity, which could have provided a more comprehensive understanding of MTX in terms of PK and clinical relevance. Future prospective studies with larger cohorts and integrated clinical data are needed to validate these findings.

Despite these limitations, our findings confirm that creatinine is the most informative biomarker for MTX exposure and clearance. Elevated creatinine predicts higher C(p), AUC_0 → 72 h_, and prolonged t½ of MTX, with age modulating this relationship at later time points. The total bilirubin may also contribute, particularly in younger patients, likely reflecting hepatic metabolic capacity. In contrast, the lipemic, icteric, and haemolysis indices do not influence MTX PK or quantification, underscoring the reliability of MTX monitoring in routine clinical practice. These results support a TDM and biomarker-driven approach to HDMTX monitoring, with emphasis on MTX C(p), MTX AUC_0 → 72 h_, renal, and hepatic function to identify patients at risk of increased exposure. Incorporating creatinine and bilirubin into adaptive dosing models may improve the safety and efficacy, especially in younger or renally compromised patients.

## 5. Conclusions

Our findings underscore the clinical relevance of incorporating renal and hepatic biomarkers into HDMTX monitoring protocols. Integrating creatinine and bilirubin assessment with TDM may facilitate the early identification of patients at risk of toxicity and support safer, individualised dosing strategies in adults with acute lymphoblastic leukaemia.

## Figures and Tables

**Table 1:** Overview of the PK parameters in patients. MTX C(p): methotrexate plasmatic concentration, AUC: area under the curve, t½: half-life; SEM: standard error of the mean; * *p* < 0.001 of MTX C(p) at 48 h compared with 24 h; ** *p* < 0.001 of MTX C(p) at 72 h compared with 24 and 48 h, §§ *p* < 0.001 of MTX t½ of second cycle compared with the first cycle in males.

PK Parameters	All Patients	Females	Males
Age (mean, range)	48.57 (19–76)	49.11 (19–72)	47.58 (19–76)
MTX C(p) 24 h (μmol/L; mean ± SEM)	36.09 (±15.53)	18.00 (±12.02)	51.93 (±26.82)
MTX C(p) 48 h	0.93 (±0.43) *	0.39 (±0.09) *	1.24 (±0.67) *
MTX C(p) 72 h	0.30 (±0.07) **	0.28 (±0.07) **	0.3 (±0.09) **
Overall AUC_0 → 72 h_ (h·μmol/L; mean ± SEM)	112.85 (±34.09)	68.77 (±26.12)	136.43 (±50.37)
AUC_0 → 72 h_ first cycle	98.48 (±34.83)	77.18 (±36.98)	110.48 (±50.50)
AUC_0 → 72 h_ second cycle	126.86 (±89.10)	46.33 (±28.42)	165.21 (±131.07)
AUC_0 → 72 h_ third cycle	237.92 (±200.64)	75.87 (±68.47)	344.74 (±336.72)
t½ (h; mean ± SEM)	17.15 (±2.40)	22.20 (±6.54)	14.45 (±1.15)
t½ first cycle	17.96 (±3.60)	26.20 (±9.57)	13.32 (±1.48)
t½ second cycle	16.09 (±2.04)	16.46 (±2.04)	15.91 (±2.46) §§
t½ third cycle	12.88 (±2.48)	10.44 (±5.23)	14.50 (±2.49)

## Data Availability

The data will be made available at reasonable request to the corresponding author.

## Abbreviations

The following abbreviations are used in this manuscript:

MTX	Methotrexate
HDMTX	High-dose methotrexate
ALL	Acute lymphoblastic leukaemia
AUC	Area under the curve
PK	Pharmacokinetics
TDM	Therapeutic drug monitoring
C(p)	Plasmatic concentration
t½	Half-life

## References

[1] Mantadakis E., Cole P.D., Kamen B.A. (2005). High-Dose Methotrexate in Acute Lymphoblastic Leukemia: Where Is the Evidence for Its Continued Use?. Pharmacotherapy.

[2] Meyer L.M., Miller F.R., Rowen M.J., Bock G., Rutzky J. (1950). Treatment of Acute Leukemia with Amethopterin (4-Amino, 10-Methyl Pteroyl Glutamic Acid). Acta Haematol..

[3] Hashkes P.J., Becker M.L., Cabral D.A., Laxer R.M., Paller A.S., Rabinovich C.E., Turner D., Zulian F. (2014). Methotrexate: New Uses for an Old Drug. J. Pediatr..

[4] Gupta K., Ravindran V. (2025). Low-Dose Methotrexate in Rheumatology: A Reinvented Drug. J. R. Coll. Physicians Edinb..

[5] Aickara D., Bashyam A.M., Pichardo R.O., Feldman S.R. (2022). Topical Methotrexate in Dermatology: A Review of the Literature. J. Dermatol. Treat..

[6] Solangon S.A., Van Wely M., Van Mello N., Mol B.W., Ross J.A., Jurkovic D. (2023). Methotrexate vs Expectant Management for Treatment of Tubal Ectopic Pregnancy: An Individual Participant Data Meta-analysis. Acta Obstet. Gynecol. Scand..

[7] Larsen E.C., Devidas M., Chen S., Salzer W.L., Raetz E.A., Loh M.L., Mattano L.A., Cole C., Eicher A., Haugan M. (2016). Dexamethasone and High-Dose Methotrexate Improve Outcome for Children and Young Adults with High-Risk B-Acute Lymphoblastic Leukemia: A Report From Children’s Oncology Group Study AALL0232. J. Clin. Oncol..

[8] Aldoss I., Forman S.J., Pullarkat V. (2019). Acute Lymphoblastic Leukemia in the Older Adult. J. Oncol. Pract..

[9] Gökbuget N., Boissel N., Chiaretti S., Dombret H., Doubek M., Fielding A., Foà R., Giebel S., Hoelzer D., Hunault M. (2024). Management of ALL in Adults: 2024 ELN Recommendations from a European Expert Panel. Blood.

[10] Howard S.C., McCormick J., Pui C.-H., Buddington R.K., Harvey R.D. (2016). Preventing and Managing Toxicities of High-Dose Methotrexate. Oncologist.

[11] Giletti A., Esperon P. (2018). Genetic Markers in Methotrexate Treatments. Pharmacogenom. J..

[12] Toksvang L.N., Brigitha L.J., van der Sluis I.M., Brivio E., Raja R., Pontoppidan P., Buhl Rasmussen A.S., Andres-Jensen L., Uhlving H.H., Kielsen K. (2025). Therapeutic Drug Monitoring in Acute Lymphoblastic Leukemia–a Deep Dive into Pharmacokinetics, -Dynamics, and -Genetics of Antileukemic Drugs. Expert. Rev. Clin. Pharmacol..

[13] Song Z., Hu Y., Liu S., Wang G., Zhai S., Zhang X., Li Y., Du G., Shi Y., Chen Y. (2022). Medication Therapy of High-dose Methotrexate: An Evidence-based Practice Guideline of the Division of Therapeutic Drug Monitoring, Chinese Pharmacological Society. Br. J. Clin. Pharmacol..

[14] Lin F., Juan Y., Zheng S.-E., Shen Z., Tang L.-N., Zhao H., Yao Y. (2009). Relationship of Serum Methotrexate Concentration in High-Dose Methotrexate Chemotherapy to Prognosis and Tolerability: A Prospective Cohort Study in Chinese Adults with Osteosarcoma. Curr. Ther. Res..

[15] Widemann B.C., Adamson P.C. (2006). Understanding and Managing Methotrexate Nephrotoxicity. Oncologist.

[16] Zhang J.C., Stotts M.J., Horton B., Schiff D. (2023). Hepatotoxicity from High-Dose Methotrexate in Primary Central Nervous System Lymphoma. Neuro-Oncol. Pract..

[17] Florin L., Lemahieu C., Stove V. (2016). Evaluation of the New Methotrexate CMIA Assay on the Architect I2000SR. Clin. Chem. Lab. Med. (CCLM).

[18] Tian G., Wu Y., Jin X., Zeng Z., Gu X., Li T., Chen X., Li G., Liu J. (2022). The Incidence Rate and Influence Factors of Hemolysis, Lipemia, Icterus in Fasting Serum Biochemistry Specimens. PLoS One.

[19] (2015). Quality Management Systems—Requirements.

[20] Zhang Y., Huo M., Zhou J., Xie S. (2010). PKSolver: An Add-in Program for Pharmacokinetic and Pharmacodynamic Data Analysis in Microsoft Excel. Comput. Methods Programs Biomed..

[21] Wickham H. (2016). Ggplot2 Graphics for Data Analysis.

[22] Takeda M., Khamdang S., Narikawa S., Kimura H., Hosoyamada M., Cha S.H., Sekine T., Endou H. (2002). Characterization of Methotrexate Transport and Its Drug Interactions with Human Organic Anion Transporters. J. Pharmacol. Exp. Ther..

[23] Kakkadath M., Naidu D., Kanthlal S.K., Sharun K. (2025). Combating Methotrexate Resistance in Cancer Treatment: A Review on Navigating Pathways and Enhancing Its Efficacy With Fat-Soluble Vitamins. Scientifica.

[24] Seydoux C., Briki M., Wagner A.D., Choong E., Guidi M., Carrara S., Thoma Y., Livio F., Girardin F.R., Marzolini C. (2024). Importance of Sex-Dependent Differences for Dosing Selection and Optimization of Chemotherapeutic Drugs. Chemotherapy.

[25] Kawakatsu S., Nikanjam M., Lin M., Le S., Saunders I., Kuo D.J., Capparelli E.V. (2019). Population Pharmacokinetic Analysis of High-Dose Methotrexate in Pediatric and Adult Oncology Patients. Cancer Chemother. Pharmacol..

[26] Balis F.M., Holcenberg J.S., Poplack D.G., Ge J., Sather H.N., Murphy R.F., Ames M.M., Waskerwitz M.J., Tubergen D.G., Zimm S. (1998). Pharmacokinetics and Pharmacodynamics of Oral Methotrexate and Mercaptopurine in Children with Lower Risk Acute Lymphoblastic Leukemia: A Joint Children’s Cancer Group and Pediatric Oncology Branch Study. Blood.

[27] Braidotti S., Zudeh G., Franca R., Kiren V., Colombini A., Bettini L.R., Brivio E., Locatelli F., Vinti L., Bertorello N. (2025). The Role of Candidate Polymorphisms in Drug Transporter Genes on High-Dose Methotrexate in the Consolidation Phase of the AIEOP-BFM ALL 2009 Protocol. Clin. Transl. Sci..

[28] Holmboe L., Andersen A.M., Mørkrid L., Slørdal L., Hall K.S. (2012). High Dose Methotrexate Chemotherapy: Pharmacokinetics, Folate and Toxicity in Osteosarcoma Patients. Br. J. Clin. Pharmacol..

[29] Lin C., Ma R., Zeng X., Zhang B., Cao T., Jiao S., Chen H., He Y., Liu M., Cai H. (2024). Integration of Genomics, Clinical Characteristics and Baseline Biological Profiles to Predict the Risk of Liver Injury Induced by High-Dose Methotrexate. Front. Pharmacol..

[30] Chen X., Li J., Yu L., Hu W., Cai J., Wang Z., Chen C., Zhang X., Xie Y., Wu K. (2024). High-dose Methotrexate Pharmacokinetics and Its Impact on Prognosis of Paediatric Acute Lymphoblastic Leukaemia Patients: A Population Pharmacokinetic Study. Br. J. Haematol..

[31] Gao X., Qian X.-W., Zhu X.-H., Yu Y., Miao H., Meng J.-H., Jiang J.-Y., Wang H.-S., Zhai X.-W. (2021). Population Pharmacokinetics of High-Dose Methotrexate in Chinese Pediatric Patients with Acute Lymphoblastic Leukemia. Front. Pharmacol..

[32] Yang Y., Wang X., Tian J., Wang Z. (2018). Renal Function and Plasma Methotrexate Concentrations Predict Toxicities in Adults Receiving High-Dose Methotrexate. Med. Sci. Monit..

[33] Tsurusawa M., Gosho M., Mori T., Mitsui T., Sunami S., Kobayashi R., Fukano R., Tanaka F., Fujita N., Inada H. (2015). Statistical Analysis of Relation between Plasma Methotrexate Concentration and Toxicity in High-dose Methotrexate Therapy of Childhood NonHodgkin Lymphoma. Pediatr. Blood Cancer.

[34] Hao Q., Song Y., Fang Q., Lin Y., Chen L., Wang X., Zhang P., Wang Z., Gong X., Liu K. (2023). Effects of Genetic Polymorphisms on Methotrexate Levels and Toxicity in Chinese Patients with Acute Lymphoblastic Leukemia. Blood Sci..

[35] Thompson P.A., Murry D.J., Rosner G.L., Lunagomez S., Blaney S.M., Berg S.L., Camitta B.M., Dreyer Z.E., Bomgaars L.R. (2007). Methotrexate Pharmacokinetics in Infants with Acute Lymphoblastic Leukemia. Cancer Chemother. Pharmacol..

[36] Kristensen L.Ø., Weismann K., Hutters L. (1975). Renal Function and the Rate of Disappearance of Methotrexate from Serum. Eur. J. Clin. Pharmacol..

[37] McTaggart M.P., Bluett J., Keevil B.G. (2024). A Sensitive LC-MS/MS Methotrexate Assay Capable of Assessing Adherence to Methotrexate Therapy in Rheumatoid Arthritis. Clin. Chem. Lab. Med. (CCLM).

[38] Li J., Rietschlin J., Miller I., Weber C., Scheidegger M., Barringer S., Kerlin R., Williams J. (2022). Evaluation of the Preanalytical Interference of Hemoglobin, Bilirubin, or Lipids in Therapeutic Drug Monitoring Assays on Beckman Coulter AU Analyzers. Lab. Med..

[39] Bouquié R., Grégoire M., Hernando H., Azoulay C., Dailly E., Monteil-Ganière C., Pineau A., Deslandes G., Jolliet P. (2016). Evaluation of a Methotrexate Chemiluminescent Microparticle Immunoassay. Am. J. Clin. Pathol..

[40] Abe K., Maeda-Minami A., Ishizu T., Iwata S., Kobayashi E., Shimoi T., Kawano Y., Hashimoto H., Yamaguchi M., Furukawa T. (2022). Risk Factors for Hepatic Toxicity of High-Dose Methotrexate in Patients with Osteosarcoma. Anticancer Res..

[41] Moghadam A.R., Tutunchi S., Namvaran-Abbas-Abad A., Yazdi M., Bonyadi F., Mohajeri D., Mazani M., Marzban H., Łos M.J., Ghavami S. (2015). Pre-Administration of Turmeric Prevents Methotrexate-Induced Liver Toxicity and Oxidative Stress. BMC Complement. Altern. Med..

[42] He X., Yao P., Li M., Liang H., Liu Y., Du S., Zhang M., Sun W., Wang Z., Hao X. (2021). A Risk Scoring Model for High-Dose Methotrexate-Induced Liver Injury in Children with Acute Lymphoblastic Leukemia Based on Gene Polymorphism Study. Front. Pharmacol..

